# Evolution and structural variations in chloroplast tRNAs in gymnosperms

**DOI:** 10.1186/s12864-021-08058-3

**Published:** 2021-10-18

**Authors:** Yu-He Zhao, Tong Zhou, Jiu-Xia Wang, Yan Li, Min-Feng Fang, Jian-Ni Liu, Zhong-Hu Li

**Affiliations:** 1 Key Laboratory of Resource Biology and Biotechnology in Western China (Ministry of Education), College of Life Sciences, Northwest University, Xi’an, 710069 China; 2 State Key Laboratory of Continental Dynamics, Department of Geology, Early Life Institute, Northwest University, Xi’an, 710069 China

**Keywords:** Chloroplast tRNA, Conservation, Evolution, Minimum free energy, Phylogenetic relationship

## Abstract

**Background:**

Chloroplast transfer RNAs (tRNAs) can participate in various vital processes. Gymnosperms have important ecological and economic value, and they are the dominant species in forest ecosystems in the Northern Hemisphere. However, the evolution and structural changes in chloroplast tRNAs in gymnosperms remain largely unclear.

**Results:**

In this study, we determined the nucleotide evolution, phylogenetic relationships, and structural variations in 1779 chloroplast tRNAs in gymnosperms. The numbers and types of tRNA genes present in the chloroplast genomes of different gymnosperms did not differ greatly, where the average number of tRNAs was 33 and the frequencies of occurrence for various types of tRNAs were generally consistent. Nearly half of the anticodons were absent. Molecular sequence variation analysis identified the conserved secondary structures of tRNAs. About a quarter of the tRNA genes were found to contain precoded 3′ CCA tails. A few tRNAs have undergone novel structural changes that are closely related to their minimum free energy, and these structural changes affect the stability of the tRNAs. Phylogenetic analysis showed that tRNAs have evolved from multiple common ancestors. The transition rate was higher than the transversion rate in gymnosperm chloroplast tRNAs. More loss events than duplication events have occurred in gymnosperm chloroplast tRNAs during their evolutionary process.

**Conclusions:**

These findings provide novel insights into the molecular evolution and biological characteristics of chloroplast tRNAs in gymnosperms.

**Supplementary Information:**

The online version contains supplementary material available at 10.1186/s12864-021-08058-3.

## Background

Gymnosperms comprise a large group of seed plants with a widespread distribution around the world. Gymnosperms are the dominant species that form forest ecosystems in the Northern Hemisphere, which constitute 39% of the world’s forests, and they have great ecological and economic significance [1]. According to the Christenhusz gymnosperms system, the extant gymnosperms are divided into 12 families, 86 genera, and about 1063 species [2]. Conifers are the most abundant group of existing gymnosperms, and they occupy a similar niche to that in the early stages of their evolution because they have strong drought resistance [3]. The genetic relationships between gymnosperms and angiosperms mean that their phylogenetic status is important [4]. Furthermore, gymnosperms have a long and extensive fossil record that dates back to the Carboniferous (c. 290 million years ago (Mya)). The five main lineages of gymnosperms (cycads, Ginkgos, cupressophytes, Pinaceae, and gnetophytes) separated from each other during the Late Carboniferous to the Late Triassic (311–212 Mya) [5].

Transfer RNA (tRNA) is one of the most ancestral types of RNA and tRNAs are ubiquitous in all living organisms from prokaryotes to eukaryotes [6]. tRNAs comprise a class of microRNAs that carry and transport amino acids, and they play central roles as the links between mRNA and protein. During protein translation, a tRNA pairs its anticodon with a codon on mRNA and carries specific amino acids to ribosome sites to mediate protein biosynthesis [7, 8]. tRNAs are multifunctional molecules that are involved in multiple metabolic processes in cells in addition to their translation function, e.g., aminoacyl-tRNA is a biosynthetic precursor and amino acid donor for other macromolecules [9]. Each tRNA can carry only one amino acid, but one amino acid can be carried by multiple tRNAs called isoreceptor tRNAs [10]. In 1965, the first tRNA comprising tRNA^Ala^ in yeast was sequenced to determine its primary structure [11]. The secondary structures of tRNAs are mostly conserved and clover-shaped, where they have an amino acid receiving arm, D-arm, anticodon arm, D-loop (a loop coupled to the D-arm), anticodon loop (a loop coupled to the anticodon arm), and a TΨC loop (a loop coupled to the TΨC arm) [12]. The nucleotide sequence of a tRNA is hydrogen bonded to form a clover-shaped secondary structure, which then folds into an inverted L-type tertiary structure [13].

Chloroplasts are multi-copy organelles in plant cells that are responsible for photosynthesis and carbohydrate metabolism [14]. Chloroplasts play vital roles in the growth and development of plants, including the synthesis of nucleotides, amino acids, fatty acids, vitamins, phytohormones, and several other metabolites [15–17]. The chloroplast genome is a highly conserved, double-stranded circular molecule containing genes that encode tRNAs, rRNAs, and many proteins [18, 19]. The semi-autonomous and complete expression system of the plant plastid genome makes it a good material for evolutionary and genomics research [20, 21]. In addition, tRNAs act as a bridge in the gene expression process. Therefore, analyzing the tRNA genes in chloroplasts can provide a theoretical basis to facilitate further studies of the structure, function, and evolutionary relationship of tRNAs.

Previous studies have investigated the evolution and structure of tRNAs in several gymnosperms, Adoxaceae plants, and *Oryza sativa* [22–24]. In the present study, we selected 54 species belonging to 54 different genera in the gymnosperm phyla and systematically analyzed their chloroplast tRNAs. We extracted and re-annotated tRNA genes in the chloroplast genome of each species to determine the differences in the composition, conservation, and structural changes in chloroplast tRNAs in different plants, as well as the evolutionary relationships and main events that affected tRNAs during their evolutionary process. In addition, the relationships between the structure of gymnosperm chloroplast tRNAs and their minimum free energy were studied for the first time. This main aims of this study were to understand: (1) the distributions and conservation of different types of tRNAs in gymnosperm chloroplasts; (2) why certain tRNAs always contain precoded 3′ CCA tails; (3) how the minimum free energy affects the stability of the secondary structure of tRNAs; and (4) the main types of events that have occurred in gymnosperm chloroplast tRNAs during their evolutionary history.

## Results

### Chloroplast tRNA gene compositions in gymnosperms

**Fig. 1:**
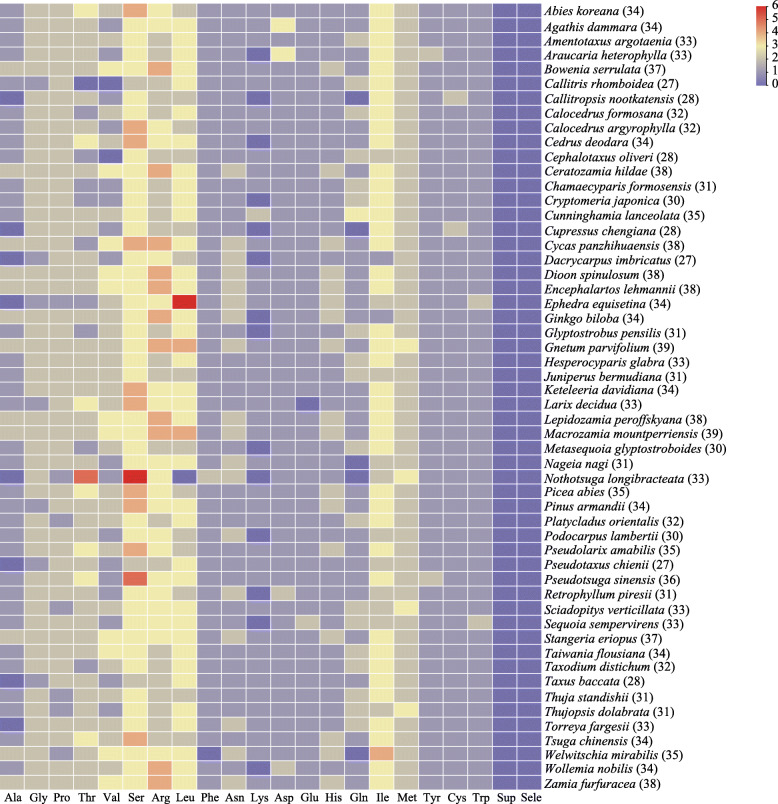
Heatmap of the distribution frequency of tRNA genes in 54 gymnosperm chloroplast genomes. The row names are the gymnosperm species, and the total numbers of tRNA genes in the chloroplast genomes of each species are in parentheses. The column names are the types of tRNAs

In the chloroplast genomes of the 54 gymnosperms considered in this study (Table S1), 1779 tRNA genes were annotated that encoded 20 essential amino acids. The chloroplast tRNA gene contents of the plants were relatively uniform [8]. The average number of chloroplast tRNA genes in each species was approximately 33. *Callitris rhomboidea*, *Dacrycarpus imbricatus*, and *Pseudotaxus chienii* encoded only 27 tRNAs, and *Gnetum parvifolium* and *Macrozamia mountperriensis* encoded up to 39 tRNAs (Fig. 1).

Almost every tRNA was encoded in the chloroplast genome of each species, but some tRNAs were not encoded in some species (Fig. 1). In particular, tRNA^Ala^ was found to be missing in eight species, tRNA^Thr^, tRNA^Glu^, tRNA^Phe^, and tRNA^Leu^ were missing in one species, tRNA^Val^ was missing in two species, tRNA^Lys^ was missing in 14 species, and tRNA^Gln^ was missing in five species. More tRNA^Ser^, tRNA^Arg^, and tRNA^Leu^ genes were present in the chloroplast genomes of all species. tRNA^Ser^ appeared three times in most species, two or four times in some species, and six times in *Nothotsuga longibracteata*. tRNA^Arg^ and tRNA^Leu^ generally appeared 2–3 times in many species, but tRNA^Leu^ appeared six times in *Ephedra equisetina*. tRNA^Gly^, tRNA^Pro^, and tRNA^Thr^ were the next most abundant tRNA genes and they occurred twice in most species. However, suppressor tRNA and selenocysteine were completely absent from the chloroplast genomes of the 54 gymnosperms, as also found in Adoxaceae [23] and monocot plants [24].

The lengths of the gymnosperm chloroplast tRNAs ranged from 56 to 90 nucleotides, and the average length was about 82 nucleotides. tRNA^Gly^ (UCC) in *Cunninghamia lanceolata* was the smallest gene detected and it only contained 56 nucleotides. The sequences of tRNA^Leu^, tRNA^Ser^, and tRNA^Tyr^ all contained more than 80 nucleotides. A few tRNA^Ser^ genes contained 90 nucleotides, and tRNA^Gly^ (UCC) in *Sequoia sempervirens* was also 90 nucleotides in length. The lengths of the other tRNAs were all about 73 nucleotides, but a few were shorter than 70 nucleotides.

### Gymnosperm chloroplast tRNAs contain 34 anticodons

The genetic code is degenerate and the 20 amino acids are encoded by 61 triplet codes [25]. However, we found that the gymnosperm tRNAs contained 34 different anticodons in 1779 tRNAs and the remaining 27 anticodons were not found in any of the tRNAs in the gymnosperm chloroplast genomes investigated in this study (Table 1). The anticodons determined in this study are as follows: tRNA^Ala^ (UGC), tRNA^Gly^ (GCC and UCC), tRNA^Pro^ (GGG and UGG), tRNA^Thr^ (GGU and UGU), tRNA^Val^ (GAC and UAC), tRNA^Ser^ (GGA, UGA, and GCU), tRNA^Arg^ (ACG, CCG, and UCU), tRNA^Leu^ (GAG, UAG, CAA, and UAA), tRNA^Phe^ (GAA), tRNA^Asn^ (GUU), tRNA^Lys^ (UUU), tRNA^Asp^ (GUC), tRNA^Glu^ (UUC), tRNA^His^ (GUG), tRNA^Gln^ (UUG), tRNA^Ile^ (GAU and CAU), tRNA^Met^ (CAU), tRNA^Tyr^ (GUA), tRNA^Cys^ (GCA), and tRNA^Trp^ (CCA). In particular, tRNA^Leu^ had the highest abundance of isoreceptors (GAG, UAG, CAA, and UAA), followed by tRNA^Ser^ (GGA, UGA, and GCU), tRNA^Arg^ (ACG, CCG, and UCU) and tRNA^Ile^ (GAU, CAU, and UAU). In addition, tRNA^Leu^ (GAG) was present only in *Ephedra equisetina*. tRNA^Lys^ (CUU) was present only in *Cunninghamia lanceolata*. tRNA^Ile^ (UAU) was present only in *Taxus baccata*. tRNA^Met^ (CAU) was present at least twice in each species. tRNA^Gly^ (GCC), tRNA^Pro^ (UGG), tRNA^Ser^ (UGA, GCU), tRNA^Arg^ (ACG), tRNA^Asn^ (GUU), tRNA^Asp^ (GUC), tRNA^His^ (GUG), tRNA^Ile^ (CAU), tRNA^Met^ (CAU), tRNA^Tyr^ (GUA), and tRNA^Trp^ (CCA) were present in all of the gymnosperm chloroplast genomes investigated in this study.

**Table 1:** Distribution of anticodons in the chloroplast genomes of gymnosperms

tRNA	Anticodon					
Alanine	AGC (0)	GGC (0)	CGC (0)	UGC (46)		
Glycine	ACC (0)	GCC (54)	CCC (0)	UCC (46)		
Proline	AGG (0)	GGG (47)	CGG (0)	UGG (54)		
Threonine	AGU (0)	GGU (46)	CGU (0)	UGU (51)		
Valine	AAC (0)	GAC (47)	CAC (0)	UAC (36)		
Serine	AGA (0)	GGA (50)	CGA (0)	UGA (54)	ACU (0)	GCU (54)
Arginine	ACG (54)	GCG (0)	CCG (28)	UCG (0)	CCU (0)	UCU (53)
Leucine	AAG (0)	GAG (2)	CAG (0)	UAG (45)	CAA (51)	UAA (40)
Phenylalanine	AAA (0)	GAA (53)				
Asparagine	AUU (0)	GUU (54)				
Lysine	CUU (0)	UUU (40)				
Aspartate	AUC (0)	GUC (54)				
Glutamate	CUC (0)	UUC (53)				
Histidine	AUG (0)	GUG (54)				
Glutamine	CUG (0)	UUG (49)				
Isoleucine	AAU (0)	GAU (47)	CAU (54)	UAU (0)		
Methionine	CAU (54)	AUA (0)				
Tyrosine	GUA (54)	ACA (0)				
Cysteine	GCA (53)					
Tryptophan	CCA (54)					

### Conservation of gymnosperm chloroplast tRNAs

Different tRNAs can transport different amino acids according to their nucleotide compositions and structures. The tRNA sequences were analyzed to identify their conserved regions (Table 2). Comparative analysis of the nucleotide compositions in the tRNA loops and arms detected conserved nucleotides or nucleotide sequences in multiple positions. In particular, these analyses showed that at the first position in the acceptor arm, tRNA^Ala^ (UGC), tRNA^Gly^ (GCC and UCC), tRNA^Thr^ (GGU), tRNA^Ser^ (GCU, GGA, and UGA), tRNA^Arg^ (ACG and CCG), tRNA^Leu^ (CAA, GAG, UAA, and UAG), tRNA^Lys^ (UUU), tRNA^Phe^ (GAA), tRNA^Asp^ (GUC), tRNA^Glu^ (UUC), tRNA^His^ (GUG), tRNA^Ile^ (CAU, GAU), tRNA^Tyr^ (GUA), and tRNA^Cys^ (GCA) contained a conserved 5′ G nucleotide, whereas tRNA^Pro^ (UGG), tRNA^Met^ (CAU), and tRNA^Val^ (GAC and UAC) contain a conserved A nucleotide, and tRNA^Asn^ (GUU) and tRNA^Gln^ (UUG) contained a U nucleotide. However, the nucleotide in the first position in the acceptor arm was not highly conserved in tRNA^Trp^ (CCA), tRNA^fMet^ (CAU), and tRNA^Thr^ (UGU). The G nucleotide content was higher in the region of the acceptor arm. tRNA^Ser^ (GCU and UGA) had conserved G-G-A-G-A-G-A nucleotide sequences in the acceptor arm. In the first position in the D-arm, tRNA^Val^ (GAC) and tRNA^Lys^ (UUU) contained a conserved A nucleotide, tRNA^Tyr^ (GUA) contained a conserved C nucleotide, and tRNA^Pro^ (GGG) and tRNA^Thr^ (GGU) contained an A or G nucleotide, whereas tRNA^Met^ (CAU) contained no conserved nucleotides in this position, and all of the other tRNAs contained a conserved G nucleotide. In addition, tRNA^Ala^ (UGC), tRNA^Thr^ (UGU), tRNA^Val^ (UAC), tRNA^Arg^ (ACG and CCG), tRNA^Leu^ (GAG), tRNA^Phe^ (GAA), tRNA^Asn^ (GUU), and tRNA^Ile^ (GAU) contained a conserved G-C-U-C nucleotide sequence, and tRNA^Cys^ (GCA), tRNA^His^ (GUG), and tRNA^Gln^ (UUG) contained a conserved G-C-C nucleotide sequence. The D-loop was found to contain a conserved A nucleotide in the first position, except in tRNA^Gly^ (GCC), tRNA^Ser^ (GCU and GGA), tRNA^Leu^ (UAA), and tRNA^Ile^ (CAU). The last position in the D-loop comprised a highly conserved A nucleotide, except in tRNA^Gly^ (GCC). The degree of conservation was lower in the anticodon arms with no conserved nucleotides in any position (Table 2). The second position in the anticodon loop was a conserved T nucleotide. The last position in the anticodon loop was generally a conserved A nucleotide. In addition, it should be noted uracil and adenine were strongly preferred in the anticodon loop. Moreover, the conservation of nucleotides was very low in the variable region because of its structural variability, although many tRNAs still possessed a conserved C nucleotide in the last position in the variable region. The Ψ-arm and Ψ-loop were the most highly conserved regions in terms of both the nucleotide number and nucleotide composition. The Ψ-arms all contained five nucleotides in the last two positions in this region, and they were mostly G nucleotides in the tRNAs. The Ψ-loops all contained seven nucleotides with a highly conserved U-U-C sequence and most tRNAs had a conserved U nucleotide in the last position.

**Table 2:** Conserved nucleotides in gymnosperm chloroplast tRNAs. AC arm: acceptor arm; ANC arm: anticodon arm; ANC loop: anticodon loop; Ψ-arm: pseudouridine arm; Ψ-loop: pseudouridine loop

tRNA Isotype	Anticodon	AC arm	D-arm	D-loop	ANC arm	ANC loop	Variable region	Ψ-arm	Ψ-loop
Ala	UGC	G-G-G-G-A-U-A	G-C-U-C	A-G-U-U-G-G-U-A	C-C-G-C-U	C-U-U-G-C-A-A	A-U-G-U-C	A-G-C-G-G	U-U-C-G-A-G-U
Gly	GCC	G-C-G-G-G-U-A	G-U-X_0–1_	G_0–1_-A-A-U-G-G-U-A-X_0–1_	U-C-U-C-C/U	U-U-G-C-C-A-A/G	A-G-A-U	G-C-G-G-G	U-U-C-G-A-U-C/U
	UCC	G-C-G-G-G-U-A	G-U-U-U	A-G-U-G-G-U-A	U-A-G-C	C-U-U-C-C-A-A	A-C-G-A-U	G-C-G-G-G	U-U-C-G-A-U-U
Pro	GGG	C-G-G-A/G-G-U/C-A	A/G-C-G-C	A-G-C/U-U-U-G-G-U-A	C-C-A-U-C	U-U-G-G-G-G-U	A-A/G-G-U-C	G-C/U-G-G-G	U-U-C-A-A/G-A-U
	UGG	A-G-G-G-A-U-G	G-C-G-C	A-G-C-U-U-G-G-U-A	U-U-U-G-U	U-U-U-G-G-G-U	A-U-G-U-C	G-C-G/A-G-G	U-U-C-A-A-A-U
Thr	GGU	G_0–1_-C-C-C-U-U-U	A/G-C-U-C	A-G-X-G-G-G-U-A	A-X-G-C-C	A-U-G-G-U-A-A	A-G-G-U-C	A-U-C-G-G	U-U-C-A-A-A/G-U/C
	UGU	X_0–1_-G-C-C-U/C-G-C-U	G-C-U-C	A-G-A-G-G-U-U/G-A	U_0–1_-C-G-C-A	C-U-U-G-U-A-A	U/C-G-G-U-C	A-U-C-G-G	U-U-C-G-A-U-U/C
Ser	GCU	G-G-A-G-A-G-A	G-C-U	G-A-G-X-G-G-A-C/U-C/U-A-A	G-U/C-G-G-A	U-U-G-C-U-A-A	G-U-A-C-X_2–3_	G-A-G-G-G	U-U-C-G-A-A-U
	GGA	G-G-A-A-A-G-A	G-C-C/U	G-A-G-C/U-G-G-U-U-C-A-A	U-A-G-C-A	U-U-G-G-A-A-C	G-U-A-G-X-C/U	G-A/G-G-G-G	U-U-C-G-A-A-U
	UGA	G-G-A-G-A-G-A	G-C-C-G	A-G-U-G-G-U-U-X-A	C-C/U-G-G-U	C-U-U-G-A-A-A	A-U-A-G-X_0–2_	G-A-G-G-G	U-U-C-A/G-A-A-U
Val	GAC	A-G-G-G-A-U-A	A-C-U-C	A-G-C/U-G-G-U/G-A	U-C-A-C-C/U	U-U-G-A-C-X-U	U/A-A-G-U-C	A-U-C-A/G-G	U-U-C-G/A-A-X_2_
	UAC	A-G-G-G-C-U-A	G-C-U-C	A-G-C-X_1–2_-G-G-U-A	C-U-C-G	U-U-U-A-C-A-C-X_0–2_	A-A-G-G-U-C	U-A-C-G-G	U-U-C-G-A-G-C/U
Arg	ACG	G-G-G-C-C-U-G	G-C-U-C	A-G-A-G-G-A-U-X-A	C-G-U-G-G	C/U-U-A-C-G-A-A	G-U-G-U-C	G-G-G-G-G	U-U-C-A/G-A-A-U
	CCG	G-G-G-U-U-A/G-G	G-C-U-C	A-G-U-G-G-A-U-C-A	C-A-U-G-G	U-U/C-C-C-G-G-A	G/A-G/A-G-U/C-C	A-A-G-G-G	U-U-C-A/G-A-A-U
	UCU	G/A-C-G-U-C-C-A	G-U-C-U	A-A-U-G-G-A-U/A-A	G-A/G-G-G-U	C-U-U-C-U-A-A	X_2_-G-U	A-U-A-G-G	U-U-C-A/G-A-A-U
Leu	CAA	G-C-C-U-U-G-A	G-U-G	G/A-A-A-U-G-G-U-A-G-A	C-G-A-G/C-A_0–1_	X_1–2_-U-C-A-A-A-A-X_0–1_	G-C-U	G/A-G-A-G-G	U-U-C-G-A-A/G-U
	GAG	G-G-G-C-U-A-U	G-C-U-C	A-G-C-G-G-U-A	U-G-C-C-C	C-U-G-A-G-A-A	G-G-G-U-C	U-C-U-G-G	U-U-C-A-A-G-U
	UAA	G-G-G-G-A-U-A	G-C-G	G-A-A-U-U/C-G-G-U-A-G/C-A	A-C-G-G-A	C-U-U-A-A-A-A	X_2–15_-U	G-A-G-G-G	U-U-C-A/G-A-A/G-U
	UAG	G-C-C-G-C-C-A	G-U-G	A-A-A-U-U/A-G-G-U-A-G-A	C-C/U-G-C/U-U	C-U-U-A-G-G-X	X_0–1_-G-C-X_8_	C-U-C-G-G	U-U-C-A/G-A-A-U
Lys	UUU	G-U-U-G-X	A-C-U-C	A-A-U-G-G-U/C-A	U-C-G-G	C-U-U-U-U-A-A	X_2_-A-G-C/U-U	C-C-G-G-G	U-U-C-A/G-A-A/G-U
Phe	GAA	G-C-C-G-G-G-A	G-C-U-C	A-G-U-U-G-G-U-A	G-A-G-G-A	C-U-G-A-A-A-A	G-U-G-C/U-C	A-C-C-A-G	U-U-C-A-A-X-U
Asn	GUU	U-C-C-U/C-C-A-A/G	G-C-U-C	A-G-X-G-G-U-A	G-U-C-G-G	C-U-G-U-U-A-A	U-A/G-G-U-C	G-U-A-G-G	U-U-C-A/G-A-A-U
Asp	GUC	G-G-G-A-U-U-G	G-U-U-C	A-A-U-U-G-G-U-C/U-A	C-C-G-C/U-C	C-U-G-U-C-A-A	A-A-G-U-U	G/A-C-G-G-G	U-U-C-G-A-G-C/U
Glu	UUC	G-C-C-C-C-U-A	G-U-C-U	A-G-U-G-G-X-C-C-A	U-C-U-C-U	C-U-U-U-C-A-A	C-A-A/G-C	G-G-G-G-A	U-U-C-G-A-X-U
His	GUG	G-X-G-G-A-C-G	G-C-C	A-A-G-U-G-G-X_2–4_-A-A	G-U-G-G-A	U-U-G-U-G-A-A	C-A-C-G/A-C	G-C-G-G-G	U-U-C-A/G-A-U-C/U
Gln	UUG	U-G-G-G-G-C/U-G	G-C-C	A-A-G-C/U-G-G-U-A-A	A/G-C-A/G-G-G	U-U-U-U-G-A/G-U	U-A-X_2–3_-C	G-A/G-A-G-G	U-U-C-G-A-A-U
fMet	CAU	X_0–1_-G-C-G-G-A/G-G	G-A-G-C/U	A-G-U/C-U/C-U-G-G-U-A	C-A/G-A-G-G	C-U-C-A-U-A-A	A-A/G-G-X-C	A-C/U-G-G-G	U-U-C-A-A-A-U
Met	CAU	A/G-C-C/A-C/U-A-C-U/A	X-C-U-C	A-A/G-U-G-G-U/G-U-A	U/C-C-X_3_	C/U-U-C-A-U-A-A/C	G/A-A/G-G-U-C	X-U-U-G-G	U-U-C-A-A-X-U
Ile	CAU	G-C/U-A-U-C-C/U-A	G-C-U	G-A-A-U/C-G-G-U-U/A-A-A	C-C-C-A-A	C-U-C-A-U-A-A	A-A-X-U-C	G-C-A/G-G-G	U-U-C-A-A-U-U
	GAU	G-G-G-C-U-X_2_	G-C-U-C	A-G-C/U-G-G-U-A	C-G-C-C-C	C-U-X-A-U-A-A	A-G-G-U/A-C	X-C-X-G-G	U-U-C-A-A-X_2_
Tyr	GUA	G-G-G-U-C-G-A	C-C-C-G	A-G-U-G-G-C/U-U-A/C-A	A-C-G-G-X	X-U-G-U-A-A-A	G-G-X_1–14_	G-C-U/A-G/A-G/U	U-U-C-A-A-A-U
Cys	GCA	G-G-X-G-A/G-C/U-A	G-C-C	A-A-G-U/C-G-G-U-A-A	G/C-A/G-A/G-G-A	C-U-G-C-A-A-A	X-A-U-C	C-C-C-A-G	U-U-C-A/G-A-A-U
Trp	CCA	X_2_-G-C-U-C-U	G-U-U-X	A-G-X_3_-G-G-U-A	X_2_-G-G-U	C-U-C-C-A-A-A	A-U-G-U/C-C	G-U-A-G-G	U-U-C-A-A-A-U

**Fig. 2:**
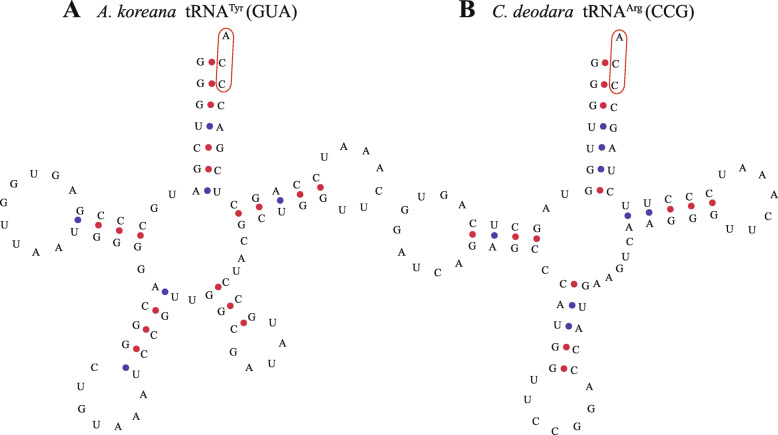
tRNAs with precoded 3′ CCA tails (marked with a red box). **(A)** tRNA^Tyr^ (GUA) in *Abies koreana*. **(B)** tRNA^Arg^ (CCG) in *Cedrus deodara*

The presence of an intact CCA sequence is a basic prerequisite for the participation of tRNAs in the mRNA decoding process [26]. The 3′ terminal regions of eukaryotic tRNAs generally lack a CCA sequence, and thus adding a 3′ CCA tail is an important step in tRNA biosynthesis. In the gymnosperms investigated in the present study, tRNA^Ala^, tRNA^Arg^, tRNA^Glu^, tRNA^Leu^, tRNA^Tyr^, and tRNA^Lys^ were found to contain a 3′ CCA tail (Fig. 2), but most tRNAs did not have 3′ CCA tails.

### Nucleotide variations in tRNA arms and loops

The number of nucleotides was also conserved in the loop arm of each tRNA. In the 1779 tRNAs considered in this study, the number of nucleotides in the acceptor arm ranged from 0 to 8 (Table 3). The acceptor arms usually contained seven nucleotides (93.25%), but 58 (3.26%) of the tRNAs contained six nucleotides in the acceptor arm. The D-arm contained three (34.23%) or four (65.65%) nucleotides in most tRNAs. However, two tRNAs had a specific D-arm that contained only one nucleotide, and both were in *Pseudotsuga sinensis* var. *wilsoniana*. The D-loops contained six to 26 nucleotides. In the 1779 tRNAs, 341 (19.17%) of the D-loops contained seven nucleotides, 281 (15.8%) contained eight, 719 (40.42%) contained nine, 162 (9.11%) contained 10, 249 (14.00%) contained 11, 25 (1.41%) contained 12, one contained six, and one contained 26 nucleotides. The anticodon arm contained four or five nucleotides, and none of the tRNAs had less than four or more than five nucleotides in the anticodon arm. We found that 97.98% of the anticodon loops contained seven nucleotides and the others had nine, 10, or 12 nucleotides. The number of nucleotides differed significantly in the variable region, where most (1049, 58.97%) contained five nucleotides, but some contained one (0.17%), two (0.39%), three (5.45%), four (15.91%), six (12.65%), seven (3.54%), eight (0.06%), 11 (2.08%), 15 (0.06%), 16 (0.73%), 17 (0.06%), or 20 (0.06%). Among all 1779 tRNAs, only one tRNA^Met^ had six nucleotides in the Ψ-arm, 11 (0.62%) contained four, and the remaining tRNAs contained five nucleotides. All tRNAs possessed seven nucleotides in the Ψ-loop.

**Table 3:** Nucleotide compositions of acceptor (AC) arm, D-arm, D-loop, anticodon (ANC) arm, anticodon loop, variable region, Ψ-arm, and Ψ-loop in chloroplast tRNAs

Region of tRNA		Nucleotide composition												
**AC arm**	Number of nucleotides	0	1	2	3	4	5	6	7	8				
	Number of tRNAs	3 (0.17%)	3 (0.17%)	1 (0.06%)	21 (1.18%)	18 (1.01%)	12 (0.67%)	58 (3.26%)	1659 (93.25%)	4 (0.22%)				
**D-arm**	Number of nucleotides	1	3	4										
	Number of tRNAs	2 (0.11%)	609 (34.23%)	1168 (65.65%)										
**D-loop**	Number of nucleotides	6	7	8	9	10	11	12	26					
	Number of tRNAs	1 (0.06%)	341 (19.17%)	281 (15.8%)	719 (40.42%)	162 (9.11%)	249 (14.00%)	25 (1.41%)	1 (0.06%)					
**ANC arm**	Number of nucleotides	4	5											
	Number of tRNAs	135 (7.59%)	1664 (93.54%)											
**ANC loop**	Number of nucleotides	7	9	10	12									
	Number of tRNAs	1743 (97.98%)	24 (1.35%)	2 (0.11%)	10 (0.56%)									
**Variable region**	Number of nucleotides	1	2	3	4	5	6	7	8	11	15	16	17	20
	Number of tRNAs	3 (0.17%)	7 (0.39%)	97 (5.45%)	283 (15.91%)	1049 (58.97%)	225 (12.65%)	63 (3.54%)	1 (0.06%)	37 (2.08%)	1 (0.06%)	13 (0.73%)	1 (0.06%)	1 (0.06%)
**Ψ-arm**	Number of nucleotides	4	5	6										
	Number of tRNAs	11 (0.62%)	1767 (99.33%)	1 (0.06%)										
**Ψ-loop**	Number of nucleotides	7												
	Number of tRNAs	1779 (100%)												

### Four types of structural changes in tRNAs

**Fig. 3:**
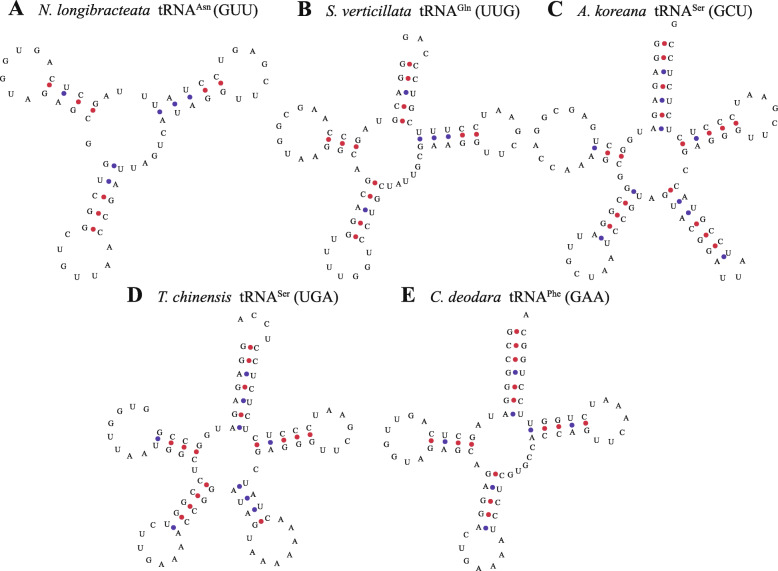
Examples of tRNAs with different structures. **(A**) Type 1 lacking an acceptor arm: tRNA^Asn^ (GUU) of *Nothotsuga longibracteata*. **(B)** Type 2 where the 3′- end contains extra nucleotides: tRNA^Gln^ (UUG) in *Sciadopitys verticillata*. **(C)** Type 3 where the variable region contains loops or arms: tRNA^Ser^ (GCU) in *Abies koreana*. **(D)** Type 4 where the 3′- end contains extra nucleotides and the variable region contains a loop and an arm: tRNA^Ser^ (UGA) in *Tsuga chinensis*. **(E)** Normal structure of tRNA^Phe^ (GAA) in *Cedrus deodara*

The general structure of a tRNA is characterized by an amino acid receiving arm, D-arm, D-loop, anticodon arm, anticodon loop, variable region loop, TΨC arm, and TΨC loop. However, some novel tRNA structures were found in the present study, which were assigned to the following four types (Table 4, Fig. 3): type 1 lacked an acceptor arm; type 2 had a 3′- end containing extra nucleotides; type 3 had a variable region containing loops or arms; and type 4 had a 3′- end containing extra nucleotides and a variable region containing a loop or arm. Among the tRNA structures with these changes, type 3 was most clearly conserved. The variable regions of tRNA^Leu^ (CAA and UAA), tRNA^Ser^ (GGA, UGA, and GCU), and tRNA^Tyr^ (GUA) had the same structure in all species, with extra loops and arms. tRNA^Leu^ also possessed a UAG anticodon, but the variable region did not have this structure. The only two tRNAs with the type 4 structure were tRNA^Tyr^ (GUA) and tRNA^Ser^ (UGA).

**Table 4:** Different structures of tRNAs and their minimum free energies

Type	Species	tRNA	Minimum free energy (kcal/mol)	Average (kcal/mol)
Type 1	*S. eriopus*	tRNA^Thr^ (GGU)	−16.0	−12.6
	*T. baccata*	tRNA^Met^ (CAU)	−12.1	
	*C. lanceolata*	tRNA^Gly^ (UCC)	−9.6	
	*N. longibracteata*	tRNA^Asn^ (GUU)	−12.8	
Type 2	*T. fargesii*	tRNA^Gly^ (UCC)	−19.5	−19.3
	*S. sempervirens*	tRNA^Gly^ (GCC)	−28.3	
	*C. nootkatensis*	tRNA^Pro^ (GGG)	−18.3	
	*C. nootkatensis*	tRNA^Ile^ (CAU)	−18.0	
	*C. oliveri*	tRNA^Met^ (CAU)	−11.8	
	*L. decidua*	tRNA^Thr^ (GGU)	−16.3	
	*P. armandii*	tRNA^Lys^ (UUU)	−20.6	
	*P. sinensis*	tRNA^Lys^ (UUU)	−20.6	
	*P. sinensis*	tRNA^Thr^ (GGU)	−20.3	
	*S. verticillata*	tRNA^Gln^ (UUG)	−21.6	
	*P. abies*	tRNA^Lys^ (UUU)	−20.6	
	*P. sinensis*	tRNA^Thr^ (GGU)	−20.3	
	*T. flousiana*	tRNA^Thr^ (GGU)	−15.1	
Type 3	*A. dammara*	tRNA^Ser^ (GCU)	−36.8	−32.8
	*A. dammara*	tRNA^Tyr^ (GUA)	−40.9	
	*A. dammara*	tRNA^Ser^ (UGA)	−30.6	
	*A. dammara*	tRNA^Ser^ (GGA)	−30.6	
	*A. dammara*	tRNA^Leu^ (UAA)	−30.2	
	*A. dammara*	tRNA^Leu^ (CAA)	−26.7	
	*A. koreana*	tRNA^Ser^ (GCU)	−38.5	
	*A. koreana*	tRNA^Ser^ (GCU)	−38.5	
	*A. koreana*	tRNA^Ser^ (UGA)	−31.6	
	*A. koreana*	tRNA^Leu^ (CAA)	−26.4	
	*A. koreana*	tRNA^Ser^ (GGA)	−35.3	
	*A. koreana*	tRNA^Tyr^ (GUA)	−33.7	
	*A. argotaenia*	tRNA^Ser^ (GCU)	−35.6	
	*A. argotaenia*	tRNA^Tyr^ (GUA)	−33.6	
	*A. argotaenia*	tRNA^Ser^ (UGA)	−32.3	
	*A. argotaenia*	tRNA^Ser^ (GGA)	−32.2	
	*A. argotaenia*	tRNA^Leu^ (UAA)	−32.9	
	*A. argotaenia*	tRNA^Leu^ (CAA)	−26.5	
	*A. heterophylla*	tRNA^Tyr^ (GUA)	−38.8	
	*A. heterophylla*	tRNA^Leu^ (CAA)	−24.9	
Type 4	*P. sinensis*	tRNA^Tyr^ (GUA)	−32.1	−28.3
	*P. sinensis*	tRNA^Ser^ (UGA)	−21.8	
	*T. chinensis*	tRNA^Ser^ (UGA)	−32.5	
	*G. biloba*	tRNA^Ser^ (UGA)	−26.6	
Normal structure	*T. flousiana*	tRNA^Lys^ (UUU)	−31.0	−26.5
	*T. flousiana*	tRNA^Gln^ (UUG)	−26.9	
	*T. flousiana*	tRNA^Gly^ (GCC)	−30.4	
	*T. baccata*	tRNA^Lys^ (UUU)	−28.1	
	*N. longibracteata*	tRNA^Glu^ (UUC)	−24.8	
	*A. dammara*	tRNA^Gln^ (UUG)	−25.8	
	*A. dammara*	tRNA^Gly^ (UCC)	−24.8	
	*A. dammara*	tRNA^Cys^ (GCA)	−27.7	
	*A. koreana*	tRNA^Pro^ (UGG)	−25.0	
	*A. koreana*	tRNA^Ile^ (CAU)	−23.5	
	*A. koreana*	tRNA^Phe^ (GAA)	−28.7	
	*A. argotaenia*	tRNA^Gly^ (UCC)	−24.8	
	*A. argotaenia*	tRNA^Arg^ (UCU)	−19.9	
	*A. argotaenia*	tRNA^Val^ (UAC)	−30.0	
	*A. argotaenia*	tRNA^His^ (GUG)	−26.4	
	*A. heterophylla*	tRNA^Cys^ (GCA)	−29.5	
	*A. heterophylla*	tRNA^Trp^ (CCA)	−23.0	
	*C. deodara*	tRNA^Gly^ (GCC)	−26.7	
	*C. oliveri*	tRNA^Pro^ (UGG)	−26.9

We calculated the minimum free energy (ΔG) for the novel tRNA and some normal tRNA structures (Table 4). The result showed that the average minimum free energy was − 12.6 kcal/mol for tRNAs with the type 1 structure, which was much higher than the normal tRNAs (ΔG = − 26.5 kcal/mol). Therefore, the absence of the acceptor arm generally reduced the stability of the tRNA structure. The minimum free energy was around − 19.3 kcal/mol for the tRNAs with the type 2 structure. tRNA^Gly^ (GCC) in *Sequoia sempervirens* had the lowest minimum free energy (ΔG = − 28.3 kcal/mol) among those with the type 2 structure, and thus the presence of extra nucleotides at the 3′ end greatly improved the stability of the structure. By contrast, tRNA^Met^ (CAU) in *Cephalotaxus oliveri* had the highest minimum free energy (ΔG = − 11.8 kcal/mol), and thus its stability was greatly reduced due to the presence of atypical nucleotides at the 3′ end. The average minimum free energy was − 33.2 kcal/mol in tRNAs with the type 3 structure. The minimum free energy values determined for these tRNAs were generally below − 30.0 kcal/mol. The values were always very low for tRNA^Tyr^ (GUA). Therefore, the loops and arms in the variable region acted together with the structures in other regions to create an extremely stable tRNA structure. However, compared with other tRNAs with the type 3 structure, tRNA^Leu^ (CAA) was remarkable because of its higher minimum free energy value of around − 26.1 kcal/mol, which was much greater than the average for tRNAs with the type 3 structure (ΔG = − 32.8 kcal/mol) and close to that for tRNAs with the normal structure (ΔG = − 26.5 kcal/mol). Thus, the structural changes in the variable region of tRNA^Leu^ (CAA) had no obvious effects. The average minimum free energy was − 28.3 kcal/mol for tRNAs with the type 4 structure and the values were quite different for each of these tRNAs, where some were above the average value and some were below. Therefore, multiple influences may have been involved when the structure changed at the 3′ end and in the variable region. Moreover, considering the average minimum free energy value for the tRNAs with normal structures (ΔG = − 26.5 kcal/mol) as a reference, the values for those with type 1 and type 2 structures were much higher, but lower for those with the type 3 structure. Thus, changes in the structures of the tRNAs affected their stability.

### Gymnosperm tRNAs evolved from multiple common ancestors

**Fig. 4:**
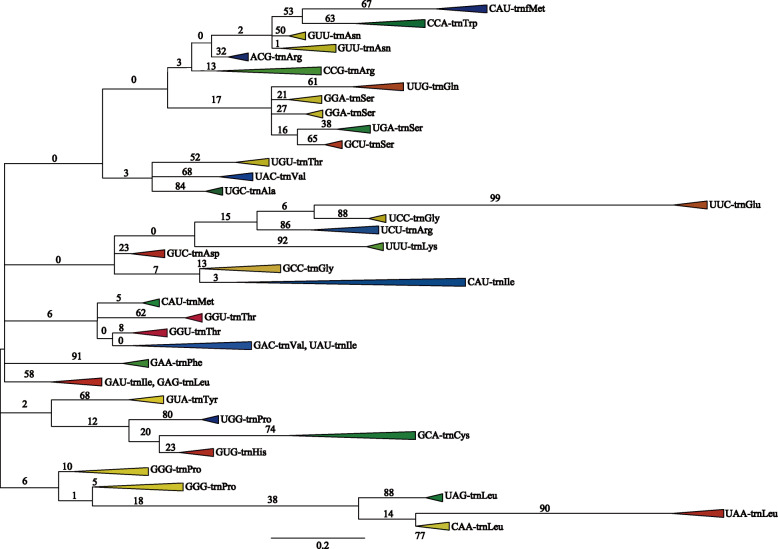
Phylogenetic relationships among all chloroplast tRNA genes in gymnosperms

In this study, the consensus coding sequences (CDSs) in the complete chloroplast genomes of 54 gymnosperms and the chloroplast genome of *Alsophila spinulosa* as an outgroup were used to construct a phylogenetic tree (Fig. S1). The result showed that species from the same family clustered on the same branch, which is consistent with the Christenhusz gymnosperms system [2] and previous studies [27]. In addition, a phylogenetic tree was constructed used the maximum likelihood method to assess the evolutionary relationships among all of the gymnosperm tRNAs (see Fig. 4 and Fig. S2, where the numbers on the branches of the evolutionary tree represent the bootstrap values). The phylogenetic tree contained two large clusters and 32 small groups. Cluster I contained 28 groups and it was much larger than cluster II with four. Not every type of anticodon was present in a group and the anticodons that occurred less frequently were often present on the same branch as other anticodons. For example, tRNA^Lys^ (CUU) appeared only once in *Cunninghamia lanceolata* and it grouped together with tRNA^Asn^ (GUU). In addition, tRNA^Ile^ (UAU) appeared only once in *Taxus baccata* and it grouped with tRNA^Val^ (GAC). Similarly, tRNA^Leu^ (GAG) appeared twice in *Ephedra equisetina* and it grouped on the branch with tRNA^Ile^ (GAU). These findings were due to the high similarity among the tRNA sequences. The low values on most branches were due to the extremely high conservation of tRNAs, where there were very few differences among the sequences.

In the top clade in the phylogenetic tree, the branches with tRNA^fMet^ (CAU) and tRNA^Trp^ (CCA), tRNA^Asn^ (GUU), tRNA^Arg^ (ACG), and tRNA^Arg^ (CCG) together indicated a stepwise evolutionary relationship. However, the other UCU anticodon of tRNA^Arg^ did not appear with tRNA^Arg^ (ACG) and tRNA^Arg^ (CCG) in 55 tRNAs, and it co-occurred with another stepwise evolutionary relationship involving tRNA^Glu^ (UUC), tRNA^Gly^ (UCC), and tRNA^Lys^ (UUU). The three tRNA^Ser^ anticodons (GGA, UGA, and GCU) occurred simultaneously in 208 tRNAs and grouped together with tRNA^Gln^ (UUG) on the same branch. These findings suggest that tRNA^Gln^ and tRNA^Ser^ belonged to a common evolutionary lineage. The three tRNA^Leu^ anticodons (CAA, UAA, and UAG) occurred simultaneously in 158 tRNAs on one branch and they were at the bottom of the phylogenetic tree. The branches containing tRNA^Leu^ and tRNA^Pro^ (GGG) together formed the second cluster. Therefore, tRNA^Pro^ and tRNA^Leu^ had a close relationship and they were far from the first cluster of tRNA groups. Moreover, tRNA^Thr^ (UGU), tRNA^Val^ (UAC), and tRNA^Ala^ (UGC) grouped together, thereby indicating their common evolutionary lineage. Similarly, the common evolutionary lineage of tRNA^Met^ (CAU), tRNA^Thr^ (GGU), and tRNA^Val^ (GAC) was evident because they were present on the same branch, and the same applied to the branch containing tRNA^Tyr^ (GUA), tRNA^Pro^ (UGG), tRNA^Cys^ (GCA), and tRNA^His^ (GUG). The phylogenetic tree also showed that tRNA^Phe^ (GAA) and tRNA^Ile^ (GAU) were grouped separately, where they each occupied a small branch instead of grouping together with the other types of tRNAs.

### Higher rate of transitions than transversions

A transition is a change from one purine to another purine (A to G or G to A) or one pyrimidine to another pyrimidine (C to U/T or U/T to C). A transversion is a change from one purine to a pyrimidine (A or G to U/T or C) or the opposite (U/T or C to A or G) [28]. Analyzing the patterns of base mutations can help to understand the molecular basis of evolution. Table 5 shows the transition and transversion rates for each tRNA as well as the overall levels in the gymnosperms investigated in the present study. tRNA^Asp^ had the highest base transition rate (25.00), while tRNA^Phe^ (22.41), tRNA^Trp^ (21.50), and tRNA^Glu^ (21.13) also had high transition rates. tRNA^His^ (11.40) had the lowest base transition rate, while tRNA^Ala^ (12.53), tRNA^Gly^ (12.61), and tRNA^Met^ (13.35) also had low transition rates. In addition, relatively high transversion rates were found for tRNA^Ala^ (6.23), tRNA^Gly^ (6.19), and tRNA^His^ (6.80), whereas tRNA^Glu^ (1.94), tRNA^Phe^ (1.29), and tRNA^Trp^ (1.75) had low transversion rates. The most remarkable group was tRNA^Asp^ with a transversion rate of zero. Overall, the transition rate was higher than the transversion rate, and the transversion rate never exceeded the transition rate in any tRNA. Similarly, we calculated the overall values in the tRNA genes and found that the transition rate (18.3) was higher than the transversion rate (3.19). Moreover, the transition rate was essentially inversely proportional to the transversion rate. Thus, when a tRNA class had a higher transition rate, it usually also had a lower transversion rate.

**Table 5:** Transition/transversion bias in gymnosperm chloroplast tRNAs

	A	U	C	G
**Alanine**				
A	–	6.23	6.23	**12.53**
U	6.23	–	**12.53**	6.23
C	6.23	**12.53**	–	6.23
G	**12.53**	6.23	6.23	–
**Asparagine**				
A	–	2.82	2.82	**19.36**
U	2.82	–	**19.36**	2.82
C	2.82	**19.36**	–	2.82
G	**19.36**	2.82	2.82	–
**Cysteine**				
A	–	2.44	2.44	**20.12**
U	2.44	–	**20.12**	2.44
C	2.44	**20.12**	–	2.44
G	**20.12**	2.44	2.44	–
**Glutamate**				
A	–	1.94	1.94	**21.13**
U	1.94	–	**21.13**	1.94
C	1.94	**21.13**	–	1.94
G	**21.13**	1.94	1.94	–
**Histidine**				
A	–	6.80	6.80	**11.40**
U	6.80	–	**11.40**	6.80
C	6.80	**11.40**	–	6.80
G	**11.40**	6.80	6.80	–
**Leucine**				
A	–	4.72	4.72	**15.56**
U	4.72	–	**15.56**	4.72
C	4.72	**15.56**	–	4.72
G	**15.56**	4.72	4.72	–
**Methionine**				
A	–	5.83	5.83	**13.35**
U	5.83	–	**13.35**	5.83
C	5.83	**13.35**	–	5.83
G	**13.35**	5.83	5.83	–
**Proline**				
A	–	2.16	2.16	**20.69**
U	2.16	–	**20.69**	2.16
C	2.16	**20.69**	–	2.16
G	**20.69**	2.16	2.16	–
**Threonine**				
A	–	3.66	3.66	**17.68**
U	3.66	–	**17.68**	3.66
C	3.66	**17.68**	–	3.66
G	**17.68**	3.66	3.66	–
**Tyrosine**				
A	–	4.04	4.04	**16.91**
U	4.04	–	**16.91**	4.04
C	4.04	**16.91**	–	4.04
G	**16.91**	4.04	4.04	–
**Arginine**				
A	–	2.48	2.48	**20.05**
U	2.48	–	**20.05**	2.48
C	2.48	**20.05**	–	2.48
G	**20.05**	2.48	2.48	–
**Aspartate**				
A	–	0.00	0.00	**25.00**
U	0.00	–	**25.00**	0.00
C	0.00	**25.00**	–	0.00
G	**25.00**	0.00	0.00	–
**Glutamine**				
A	–	4.49	4.49	**16.02**
U	4.49	–	**16.02**	4.49
C	4.49	**16.02**	–	4.49
G	**16.02**	4.49	4.49	–
**Glycine**				
A	–	6.19	6.19	**12.61**
U	6.19	–	**12.61**	6.19
C	6.19	**12.61**	–	6.19
G	**12.61**	6.19	6.19	–
**Isoleucine**				
A	–	5.08	5.08	**14.85**
U	5.08	–	**14.85**	5.08
C	5.08	**14.85**	–	5.08
G	**14.85**	5.08	5.08	–
**Lysine**				
A	–	5.37	5.37	**14.25**
U	5.37	–	**14.25**	5.37
C	5.37	**14.25**	–	5.37
G	**14.25**	5.37	5.37	–
**Phenylalanine**				
A	–	1.29	1.29	**22.41**
U	1.29	–	**22.41**	1.29
C	1.29	**22.41**	–	1.29
G	**22.41**	1.29	1.29	–
**Serine**				
A	–	4.22	4.22	**16.56**
U	4.22	–	**16.56**	4.22
C	4.22	**16.56**	–	4.22
G	**16.56**	4.22	4.22	–
**Tryptophan**				
A	–	1.75	1.75	**21.50**
U	1.75	–	**21.50**	1.75
C	1.75	**21.50**	–	1.75
G	**21.50**	1.75	1.75	–
**Valine**				
A	–	5.24	5.24	**14.52**
U	5.24	–	**14.52**	5.24
C	5.24	**14.52**	–	5.24
G	**14.52**	5.24	5.24	–
Overall				
**A**	–	**3.19**	**3.19**	18.63
**U**	**3.19**	–	18.63	**3.19**
**C**	**3.19**	18.63	–	**3.19**
**G**	18.63	**3.19**	**3.19**	–

### Duplication and loss events in gymnosperm chloroplast tRNAs

**Fig. 5:**
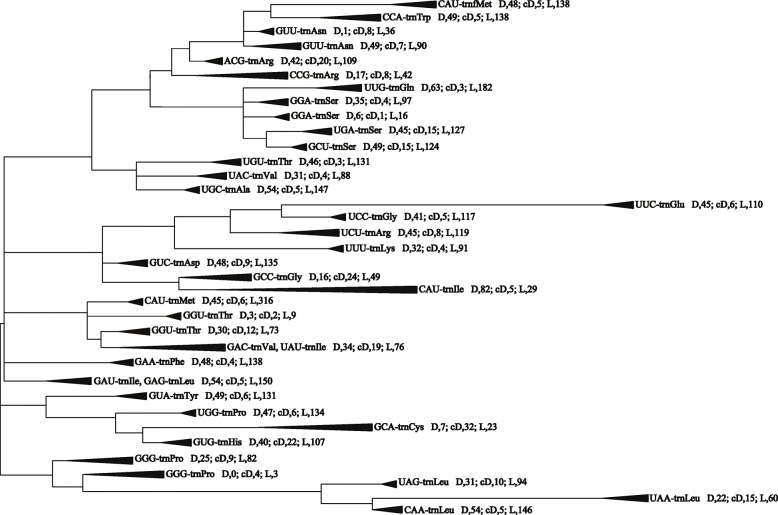
Duplication and loss events in gymnosperm chloroplast tRNAs. The results showed that gene loss events mainly occurred in tRNAs during their evolution. D, Duplication; cD, conditional duplication; L, loss

After a gene duplication event, a copy of each replicated gene pair tends to undergo a loss event. Gene loss events occur frequently [29]. We calculated the duplication and loss events in the gymnosperm chloroplast tRNAs (Fig. 5 and Fig. S3) and found that 1333 genes were duplicated whereas 3657 genes were lost. In addition, 314 genes were affected by conditional duplication events. Loss events were far more frequent than duplication events, and most of the chloroplast tRNAs had been affected by loss events during the course of their evolution.

## Discussion

### Distribution of tRNAs

Our analysis of gymnosperm chloroplast tRNAs showed that the tRNA genes were conserved in terms of both their quantity and composition. The number of tRNA genes in the chloroplast genome differed little between species and the frequency of each tRNA gene was basically the same, with only slight differences. Some tRNAs may have been lost occasionally in a few species, but tRNA genes in the nucleus or other organelles can replace the functions of these missing tRNA genes [30]. It has been shown that tRNA^Ser^, tRNA^Arg^, and tRNA^Leu^ always occur at higher frequencies in the chloroplast genomes of gymnosperms. In addition, the lengths of tRNA^Ser^ and tRNA^Leu^ sequences can clearly be longer due to the different nucleotides in the variable region. A previous study also demonstrated that tRNA^Leu^ has a large variable region [31]. The main function of the variable region in tRNAs has not been fully elucidated, but it has been shown that larger variable regions can increase the affinity of tRNA for ribosomes and stabilize the tRNA–ribosomal complex in various environments to enhance the interactions between tRNAs and ribosomes [32]. This may explain why these types of tRNAs are more commonly found in plant chloroplasts and their association with many biological processes.

Suppressor tRNA is a mutated form of tRNA and it can read mRNA in a new manner and allow the insertion of appropriate amino acids at a mutation site in a protein-coding gene to suppress the phenotypic effect of a coding mutation, thereby affecting the production of functional cellular proteins [33–36]. Suppressor tRNA is not found in gymnosperm chloroplast genomes. In addition, selenocysteine inserting tRNAs are absent from the chloroplast genomes in gymnosperms, Adoxaceae, and monocot plants [23, 24]. Selenocysteine is an atypical amino acid [37] and the 21st amino acid involved in the ribosome-mediated synthesis of proteins via a UGA codon. Selenocysteine is found in both prokaryotes and eukaryotes [38], but it is an oxygen-labile amino acid with a degree of toxicity [39]. In the present study, we found at least one tRNA^Met^ and one tRNA^fMet^ in each species, where both corresponded to the CAU anticodon. It is known that tRNA^fMet^ is necessary for initiating the protein translation process in prokaryotes [40–43]. The initiator tRNA is always well conserved [44]. tRNA^fMet^ (CAU) and tRNA^Met^ (CAU) are both essential in plants [45]. Interestingly, we found that tRNA^Met^ and tRNA^Ile^ both contained the same CAU anticodon. The relationship between the identification and matching of codons is highly complex. Previous studies in bacteria [46–48] have shown that when the C nucleotide is modified in the CAU anticodon, tRNA^Ile^ can recognize isoleucine, whereas the unmodified tRNA^Ile^ with the CAU anticodon will interact with methionine. It has also been demonstrated that this change has the same effect in plant chloroplasts [45], which can be explained by the prokaryotic origin of the chloroplast.

### Distribution of anticodons

The genetic code is based on 64 codons where 61 can encode amino acids and three are stop codons, but they usually do not all appear together. In the present study, only 34 types of anticodons were found in gymnosperm chloroplast tRNA genes, where some anticodons occurred in all species and some were only found occasionally in a few species. These 34 types of anticodons can fulfill the roles of all 61 anticodons and they are responsible for protein translation in the chloroplast. By contrast, 28 anticodons are found in the chloroplast genomes of Adoxaceae species [23] and 28 anticodons in monocot plants [24]. The degeneracy of the genetic code is explained by the “wobble hypothesis” where the first and second bases in a codon pair strongly with the anticodon but the third base can form a non-Watson–Crick base pair with the anticodon [49]. Thus, some types of tRNAs can correspond to multiple anticodon types and one amino acid can be carried by multiple tRNAs. Substitutions in protein-coding genes are usually distributed according to the codon structure and substitutions often occur at the third position in the codon. Moreover, multiple anticodons corresponding to one tRNA have the same “evolutionary potential” [25, 50]. In addition, organisms can differ in terms of their codon usage preferences. The use of synonymous codons is non-random and it is mainly determined by specific preferences in the translation process [51]. There is a strong correlation between codon usage and the tRNA content, and the codon selection pattern tends to be highly conserved in the evolutionary process. Genes with high expression levels often have codons that correspond to more abundant tRNA types [52–54], and thus the gene expression levels are strongly related to codon usage preferences [55, 56]. According to the results in Fig. 1 and Table 1, the overall frequencies of the codons contained in tRNA^Ser^, tRNA^Arg^, and tRNA^Leu^ were higher. Codon usage selectivity occurs in organisms because the use of common codons in highly abundant tRNAs can greatly reduce the risk of depleting the translation mechanism [57].

### Highly conserved secondary structure of tRNAs

The secondary structure of tRNAs is shaped like a clover leaf, with an acceptor arm containing seven nucleotides, D-arm containing 3–4 nucleotides, D-loop containing 4–12 nucleotides, anticodon arm containing five nucleotides, anticodon loop containing three nucleotides, variable region containing 4–23 nucleotides, Ψ-loop containing five nucleotides, and Ψ-arm containing seven nucleotides [58, 59]. However, we found that some of the chloroplast tRNAs had different secondary structures in gymnosperms and not all fully conformed to the traditional pattern. Moreover, the differences in the numbers of nucleotides in different tRNA regions were strongly related to the type of tRNA and they even varied according to the corresponding anticodon. For example, tRNA^Gly^ (GCC) contained four nucleotides in the variable region but tRNA^Gly^ (UCC) contained five nucleotides (Table 2). In addition to the number of nucleotides, the nucleotide compositions in different regions also varied. The sequences of tRNAs were found to be highly conserved with common nucleotides or sequences in almost every region, and their conservation was related to the type of tRNA. Alignment of the tRNA sequences showed that the Ψ-loop was the most highly conserved without any changes and the Ψ-arm was also extremely well conserved, where only a small part of the tRNA was mutated in this region. Similar results were found in a previous study of the conserved regions of chloroplast tRNAs in monocot plants [24]. The Ψ-loop contained a common sequence comprising U-U-C and it was previously reported that conserved bases in the Ψ-loop determine the stability of tRNAs in thermophilic bacteria [60]. The anticodon loops were also highly conserved where most contained seven nucleotides. The anticodon loop is the region that matches with the codon in mRNA, so high accuracy is required. The addition of a conserved C-C-A sequence at the 3′ end of tRNA is necessary for tRNA maturation, which is mediated by tRNA nucleotidyltransferase, and tRNAs can only carry amino acids when the CCA tail is present [61, 62]. However, the addition of CCA tails does not always require the action of tRNA nucleotidyltransferase, and CCA tails are sometimes included in the tRNA gene templates in bacteria. It has been reported that the templated 3′ CCA sequence in bacteria is very common in the initial tRNA (tRNA^fMet^) as well as in tRNA^Tyr^ [63]. In the present study, we found that gymnosperm plant chloroplast tRNA genes for tRNA^fMet^ and tRNA^Tyr^ all carried an encoded 3′ CCA sequence in each species, which suggests that part of the prokaryotic translation mechanism was retained during chloroplast evolution. In addition, the main factor that affects protein synthesis is the initiation of translation [64, 65], and thus 3′ CCA templating can greatly enhance the rate of protein expression because it accelerates the maturation of tRNA.

### Phylogenetic relationships

The phylogenetic analysis of all tRNA genes showed that tRNA^fMet^ (CAU) appeared twice in the phylogenetic tree, i.e., at the top of the tree grouped together with tRNA^Trp^ (CCA), and in the lower part of the tree grouped together with tRNA^Thr^ (GGU) and tRNA^Val^ (GAC) (Fig. 4 and Fig. S2). These findings indicate that tRNA^fMet^ (CAU) evolved from multiple common ancestors, and that tRNA^fMet^ (CAU) has undergone more frequent duplication events during its evolution.

### Effects of structural changes on the stability of tRNAs

The minimum free energy of a molecule is closely related to its structure and it ensures the thermodynamic stability of RNA [66]. The minimum free energy can be used to predict the secondary structure of RNA [67–69]. In thermophiles, the folding of tRNA undergoes adaptive changes to improve its stability because changes in the tertiary structure can affect the stability of tRNA [70]. In this study, we found several changes in the structure of tRNAs, which were roughly divided into four categories and clear patterns were identified in the corresponding minimum free energy values. Compared with the normal structure of tRNAs, these structural changes increased or decreased the minimum free energies of tRNAs. It has been reported that changes in the acceptor arm will increase the free energy of tRNA [71], which is consistent with the results obtained in the present study because the free energy was higher when the acceptor arm was lacking nucleotides or redundant nucleotides were present. tRNAs with large variable regions were not rare and the large variable regions have even evolved into conserved structures in some types of tRNAs, such as tRNA^Leu^. Thus, this type of structural change greatly reduced the free energy of tRNA to increase the stability of the structure. Figure 1 shows that the frequency of occurrence was relatively high for tRNA^Leu^, which may indicate that this type of structural change in tRNA^Leu^ proved beneficial for its utilization by plants.

### Evolution of substitution rate

Eight types of transversion and four types of transition are possible, and thus transversions should be more frequent from a probabilistic perspective, but our statistical results indicated a high transition rate, i.e., “transition bias” [72]. This bias can be explained by the fact that transitions have less effect on proteins than transversions [73]. In particular, conversion involves substitution with bases of the same type whereas inversion involves substitution with bases of a different type. The structural differences are small among the members within the separate purine and pyrimidine families, whereas the structural differences are large between purines and pyrimidines. Thus, transitions have less effect on the structure of proteins. In addition, the transversion rate was zero for tRNA^Asp^. One possibly because it has not undergone any transversions during its evolution. Another possibility is that the synthesis of this tRNA will be terminated if a transversion occurs in this gene, thereby resulting in an undetectable transversion rate. Overall, the chloroplast tRNAs in gymnosperms mainly underwent base transversion during their evolution.

### Duplication and loss events during evolution

Gene duplication and loss events have occurred very frequently in plant genomes, and they have been important factors during their evolution [74–76]. The size of the chloroplast genome has decreased throughout evolutionary history and gene loss events continue to occur [77]. As shown in Fig. 1 and Table 1, some tRNAs were absent from certain species and nearly half of the anticodons were also absent. These results demonstrate that loss events mainly occurred during the evolution of chloroplast tRNA genes in gymnosperms.

## Conclusions

This work provides a further explanation for the structural variations and evolution of the chloroplast tRNA in gymnosperms. We found that the chloroplast tRNAs in gymnosperms mainly underwent base transversion and loss events during their evolution. The precoded 3′ CCA sequences were found in some gymnosperm chloroplast tRNAs sequences, it suggested that part of the prokaryotic translation mechanism was retained. In addition, we speculated that the utilization of certain tRNA types in gymnosperms chloroplasts might be related to the patterns of tRNA minimum free energy.

## Materials and methods

### Acquisition of chloroplast tRNA genes and secondary structure analysis

Chloroplast genomes for 54 gymnosperms (Table S1) were downloaded from the public database at the National Center for Biotechnology Information (NCBI, https:// www.ncbi.nlm.nih.gov/). Chloroplast genome annotation and tRNA gene extraction were conducted with Geneious [78]. All of the gymnosperm chloroplast tRNA gene sequences were uploaded to the tRNAscan-Se server to predict their secondary structure and to obtain other related results [79]. The free energies of tRNAs with structural changes were calculated using the RNAalifold web server with the default parameters.

### Multiple sequence alignment

All tRNAs were classified according to their different types to identify the consensus sequence in each region. Similarly, the consensus sequence in each region was determined at the overall tRNA level. Multiple sequence alignment of tRNA genes was performed with the Multalin server [80]. All of the sequences were used for alignment analysis with the following parameters in FASTA format: sequence input format, auto; display of sequence alignment, colored; alignment matrix, Blosum61–12-2; gap penalty at opening and extension, default; gap penalty at extremities, none and one iteration only, none; highest consensus value, 90% (default); and low consensus value, 50% (default). In the displayed alignments, red indicates similarity/conservation of 90% or more, blue indicates sequence conservation less than 90%, and black indicates no conservation. The CDS sequences of chloroplast genomes in each species were obtained using Geneious and the consensus CDS sequences were then extracted. The consensus CDS sequences in the chloroplast genomes in gymnosperms and *Alsophila spinulosa* were aligned with the Linux version of MAFFT software [81].

### Phylogenetic tree construction

A phylogenetic tree was constructed using MEGA7 software to identify the phylogenetic relationships among all of the tRNAs [82]. The model with the lowest Bayesian information criterion (BIC) score was selected as the best model for constructing a phylogenetic tree. Calculations using MEGA7 software showed that the K2 + G + I model had the lowest BIC score (50,455.017), and thus it was used to construct a phylogenetic tree based on gymnosperm chloroplast tRNAs. The other parameters used to construct the phylogenetic tree were: analysis, phylogeny reconstruction; statistical model, maximum likelihood; test of phylogeny, bootstrap method; no. of bootstrap replicates, 1000; substitution type, nucleotide; rates among sites, Gamma distributed with invariant sites (G + I); no. of discrete Gamma categories, 5; gaps/missing data treatment, partial deletion; site coverage cutoff, 95%; and branch swap filter, very strong. The phylogenetic tree based on the consensus CDS sequences in the chloroplast genomes in gymnosperms and *Alsophila spinulosa* was constructed using RaxML via the CIPRES Science Gateway [83]. Node supports for the maximum likelihood analyses were estimated by performing 1000 bootstrap iterations.

### Analysis of transitions and transversions

The tRNA gene sequences used to construct the phylogenetic tree were also employed to calculate the transition and transversion rates. All of the tRNA gene sequences were classified according to their different types, before calculating the transition and transversion rates. The same calculations were performed at the overall tRNA level. The calculations were performed using MEGA7 software [82]. The following parameters were used to calculate the transition and transversion rates: analysis, substitution pattern estimation (ML); tree to use, automatic (neighbor-joining tree); statistical method, maximum likelihood; substitution type, nucleotide; model/method, Kimura2-parameter model; rates among sites, Gamma distributed (G); no. of discrete Gamma categories, 5; gaps/missing data treatment, partial deletion, site coverage cutoff 95%; and branch swap filter, very strong.

### Analysis of gene duplication and loss events

The phylogenetic trees based on the tRNA genes and species were reconciled in order to calculate duplication and loss events in tRNA genes. A species tree was constructed based on 54 gymnosperm species via the NCBI taxonomy server (https://www.ncbi.nlm.nih.gov/Taxonomy/CommonTree/wwwcmt.cgi). The species tree and gene tree were reconciled using Notung 2.9 [84].

## Supplementary Information

**Additional file 1: Table S1.** The 54 gymnosperms considered in this study and their NCBI ID numbers.

**Additional file 2: Fig. S1.** Phylogenetic tree based on the consensus CDS sequences in chloroplast genomes in gymnosperms and *Alsophila spinulosa*. ML bootstrap values are given adjacent to nodes.

**Additional file 3: Fig. S2.** Phylogenetic tree based on gymnosperm chloroplast tRNAs. The phylogenetic tree was constructed using the maximum likelihood method and 1000 bootstrap replicates with MEGA.

**Additional file 4: Fig. S3.** Duplication and loss events in gymnosperm chloroplast tRNAs. Duplication and loss analysis was conducted using the program Notung.

## Data Availability

All of the chloroplast genomic sequences used in this study can be found via the NCBI website under the accession numbers given in Table S1.

## Abbreviations

A: Adenine
C: Cytosine
G: Guanine
U: Uracil
Ψ: Pseudouridine
