# Supplementary material for: Evolution and structural variations in chloroplast tRNAs in gymnosperms
Source: BMC Genomics. 2021 Oct 18;22:750. doi: 10.1186/s12864-021-08058-3 (PMC8524817; doi:10.1186/s12864-021-08058-3)
Supplement: Supplementary file 1 — Additional file 1: Table S1. The 54 gymnosperms considered in this study and their NCBI ID numbers. [file 12864_2021_8058_MOESM1_ESM.docx]

| Table S1. 54 gymnosperms and their NCBI ID Numbers. | | |
| --- | --- | --- |
| **Genus** | **Species** | **NCBI ID** |
| Ephedra | *Ephedra equisetina* Bunge | NC_011954 |
| Gnetum | *Gnetum parvifolium* (Warb.)C. Y. Cheng ex Chun | NC_011942 |
| Welwitschia | *Welwitschia mirabilis* Hook. f. | NC_010654 |
| Cycas | *Cycas panzhihuaensis* L. Zhou & S. Y. Yang | NC_031413 |
| Stangeria | *Stangeria eriopus* (Kunze) Baill. | NC_026041 |
| Bowenia | *Bowenia serrulata* (W. Bull) Chamb. | NC_026036 |
| Dioon | *Dioon spinulosum* Dyer ex Eichler | NC_027512 |
| Encephalartos | *Encephalartos lehmannii* Lehm. | NC_027514 |
| Macrozamia | *Macrozamia mountperriensis* F. M. Bailey | NC_027511 |
| Lepidozamia | *Lepidozamia peroffskyana* Regel | NC_027513 |
| Ceratozamia | *Ceratozamia hildae* G.P.Landry & M. C. Wilson | NC_026037 |
| Zamia | *Zamia furfuracea* Ait. | NC_026040 |
| Ginkgo | *Ginkgo biloba* L. | NC_016986 |
| Pinus | *Pinus armandii* Franch. | NC_029847 |
| Picea | *Picea abies* (L.)H. Karsten | NC_021456 |
| Abies | *Abies koreana* E. H. Wilson | NC_026892 |
| Cathaya | *Cathaya argyrophylla* Chun et Kuang | NC_014589 |
| Keteleeria | *Keteleeria davidiana* (Bertr.) Beissn. | NC_011930 |
| Pseudotsuga | *Pseudotsuga sinensis* var. *wilsoniana* (Hayata) L. K. Fu & Nan Li | NC_016064 |
| Tsuga | *Tsuga chinensis* (Franch.) Pritz. | NC_030630 |
| Larix | *Larix decidua* Miller | NC_016058 |
| Pseudolarix | *Pseudolarix amabilis* (J. Nelson) Rehder | NC_030631 |
| Cedrus | *Cedrus deodara* (Roxburgh) G. Don | NC_014575 |
| Nothotsuga | *Nothotsuga longibracteata* (W. C. Cheng) Hu ex C. N. Page | NC_037407 |
| Araucaria | *Araucaria heterophylla* (Salisb.) Franco | NC_026450 |
| Agathis | *Agathis dammara* (Lamb.) Rich. et A. Rich. | NC_023119 |
| Wollemia | *Wollemia nobilis* W.G.Jones, K.D.Hill & J.M.Allen | NC_027235 |
| Podocarpus | *Podocarpus lambertii* Klotzsch ex Endl. | NC_023805 |
| Nageia | *Nageia nagi* (Thunberg) Kuntze | NC_023120 |
| Dacrycarpus | *Dacrycarpus imbricatus* (Blume) de Laubenfels | NC_034942 |
| Retrophyllum | *Retrophyllum piresii* (Silba) C.N.Page | NC_024827 |
| Sciadopitys | *Sciadopitys verticillata* (Thunb.) Sieb. et Zucc. | NC_029734 |
| Juniperus | *Juniperus bermudiana* L. | NC_024021 |
| Cupressus | *Cupressus chengiana* S. Y. Hu | NC_034788 |
| Chamaecyparis | *Chamaecyparis formosensis* Matsum. | NC_034943 |
| Hesperocyparis | *Hesperocyparis glabra* (Sudw.) Bartel | KX_832624 |
| Calocedrus | *Calocedrus formosana* (Florin) Florin | KX_832620 |
| Platycladus | *Platycladus orientalis* (L.) Franco | KX_832626 |
| Thuja | *Thuja standishii* (Gord.) Carr. | KX_832627 |
| Thujopsis | *Thujopsis dolabrata* (Thunberg ex L. f.) Sieb. et Zucc. | KX_832628 |
| Callitris | *Callitris rhomboidea* R. Br. ex Rich. & A. Rich. | NC_034940 |
| Callitropsis | *Callitropsis nootkatensis* (D.Don) Oerst. ex D.P.Little | NC_026295 |
| Cunninghamia | *Cunninghamia lanceolata* (Lamb.) Hook. | NC_021437 |
| Taiwania | *Taiwania flousiana* Gaussen | NC_021441 |
| Metasequoia | *Metasequoia glyptostroboides* Hu et W. C. Cheng | NC_027423 |
| Sequoia | *Sequoia sempervirens* (D. Don) Endl. | NC_030372 |
| Cryptomeria | *Cryptomeria japonica* (L. f.) D. Don | NC_010548 |
| Glyptostrobus | *Glyptostrobus pensilis* (Staunt. ex D. Don) K. Koch | NC_031354 |
| Taxodium | *Taxodium distichum* (L.) Rich. | NC_034941 |
| Amentotaxus | *Amentotaxus argotaenia* (Hance) Pilger | NC_027581 |
| Taxus | *Taxus baccata* L. | NC_035066 |
| Torreya | *Torreya fargesii* Franch. | NC_029398 |
| Pseudotaxus | *Pseudotaxus chienii* (W. C. Cheng) W. C. Cheng | MH_390485 |
| Cephalotaxus | *Cephalotaxus oliveri* Mast. | NC_021110 |
