# Supplementary figures and images for: Evolution and structural variations in chloroplast tRNAs in gymnosperms
Source: BMC Genomics. 2021 Oct 18;22:750. doi: 10.1186/s12864-021-08058-3 (PMC8524817; doi:10.1186/s12864-021-08058-3)

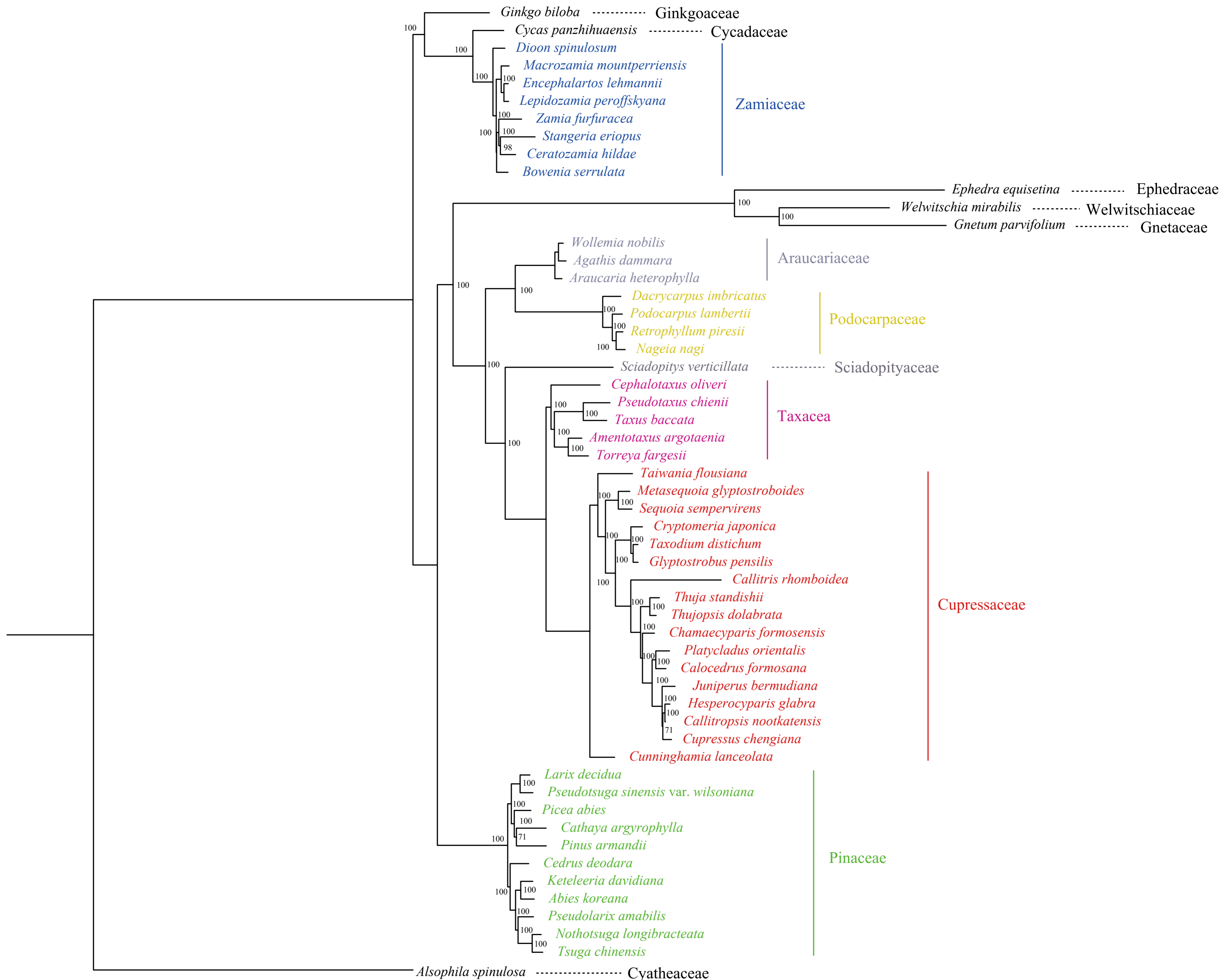

0.06

Supplement: Supplementary file 2 — Additional file 2: Fig. S1. Phylogenetic tree based on the consensus CDS sequences in chloroplast genomes in gymnosperms and Alsophila spinulosa. ML bootstrap values are given adjacent to nodes. [file 12864_2021_8058_MOESM2_ESM.pdf]
